# Fathering of Dizygotic Twins and Risk of Prostate Cancer: Nationwide, Population-Based Case-Control Study

**DOI:** 10.1371/journal.pone.0110506

**Published:** 2014-10-22

**Authors:** Sara Wirén, Linda Drevin, Olof Akre, David Robinson, Pär Stattin

**Affiliations:** 1 Department of Surgery and Perioperative Sciences, Urology and Andrology, Umeå University, Umeå, Sweden; 2 Regional Cancer Centre, Uppsala University Hospital, Uppsala, Sweden; 3 Clinical Epidemiology Unit, Department of Medicine Solna, Karolinska Institutet, Stockholm, Sweden; 4 Department of Urology, Ryhov County Hospital, Jönköping, Sweden; National Health Research Institutes, Taiwan

## Abstract

**Background:**

An association between male fertility and risk of prostate cancer has been suggested, possibly through lower androgen levels in subfertile men. We evaluated male fertility in relation to risk of prostate cancer by assessing the frequency of fathering of dizygotic twins, a marker of high fertility, among cases of prostate cancer and controls.

**Methods:**

We performed a case-control study in Prostate Cancer data Base Sweden (PCBaSe), a nationwide, population-based cohort. PCBaSe was linked to the Swedish twin register for information on zygosity for same-sex twins and to other nationwide health care registers and demographic databases for information on socioeconomic factors, comorbidity, and tumor characteristics for 96 301 prostate cancer cases and 378 583 matched controls. To account for the influence of in vitro fertilization on dizygotic twinning, analyses were restricted to men who had fathered children before 1991, when in vitro fertilization was still uncommon in Sweden.

**Results:**

1 112 cases and 4 538 controls had fathered dizygotic twins. Men with dizygotic twins had no increased risk of prostate cancer compared to fathers of singletons; neither for total prostate cancer odds ratio (OR) 0.95(95% CI 0.89–1.02), nor for any risk category, OR 0.97 (95% CI 0.84–1.12) for low-risk disease, and OR 1.04 (95% CI 0.90–1.22) for metastatic disease.

**Conclusion:**

The lack of association between fathering of dizygotic twins and prostate cancer risk give no support for an association between male fertility and prostate cancer risk.

## Introduction

Fathers have consistently been shown to have a higher risk of prostate cancer than childless men in large population-based observational studies [1]–[3]. In the most recent of these studies, using the same database as the present study, differences in marital status and educational level accounted for a part of this association [3]. However, even after adjustments for these factors, a moderately increased risk of disease in fathers remained overall and for all risk categories of prostate cancer, including metastatic disease. One possible explanation for this increased risk is through higher androgen levels in the fathers as compared to infertile men, [4], [5] as androgen levels might affect risk of prostate cancer [6]–[8].

However, there are others factors, in addition to male fertility, that affect if a man fathers children, such as fertility of the partner, presence of female partner and wish to have children. Another marker of increased fertility, fathering of dizygotic twins, should not be affected by such factors. Dizygotic twinning is considered a marker of high fertility in the couple while monozygotic twinning occur randomly and the rate of dizygotic vs monozygotic twinning has been used to monitor fertility trends on a population level [9], [10]. Also, in a study on testicular cancer, the ratio of dizygotic twinning was clearly reduced in subfertile men that later developed testicular cancer, [11] indicating that this study design can be used for studying the relationship between fertility and risk of cancer.

The aim of this study was to assess the risk of prostate cancer for men that had fathered dizygotic twins, also taking social factors, comorbidity and tumor characteristics into account.

## Materials and Methods

### The National Prostate Cancer Register of Sweden

The first regional prostate cancer register was established in south-east Sweden in 1987, and subsequentially more regions joined to form the National Prostate Cancer Register (NPCR) of Sweden [12]. NPCR is nationwide since 1998 and the capture rate is over 98% in comparison to the Swedish Cancer Register to which registration is mandatory and regulated by law. NPCR include information on diagnostic unit, date of diagnosis, tumor characteristics according to the tumor, node, metastasis (TNM) classification, data on tumor differentiation, serum level of PSA at diagnosis, and primary treatment delivered or decided within six months after diagnosis. From 2000 and onwards, data on cause for diagnostic work-up leading to the prostate cancer diagnosis are available.

### Prostate Cancer data Base Sweden (PCBaSe) 2.0

PCBaSe was created in 2008 by linkages between NPCR and a number of other nationwide demographic and health registers [13]. Linkage was performed using the unique personal identity number assigned to each Swedish resident [14]. In 2011, PCBaSe 2.0 was created through a new linkage with more cases of prostate cancer and longer follow-up [15]. By use of data in NPCR, the Longitudinal Integration Database for Health Insurance and Labor Market Studies, the Swedish Multi-Generation Register, and the National Patient Register, information was obtained on tumor characteristics, number of children, educational level and comorbidity. In Sweden, no formal screening for prostate cancer has been or is currently in operation, apart from in the Gothenburg area where 10 000 men have been invited to PSA-screening as part of a randomized trial [16].

### Identification of cases and controls

We included all cases of prostate cancer in PCBaSe 2.0 diagnosed between 1991 and 2009. For each case of prostate cancer, two controls for the period of 1991–1995 and five controls for the period 1996–2009 were randomly sampled among prostate-cancer free men in the Swedish population, matched for birth year (+/− 1 year) and county of residence. Analysis was restricted to fathers and we thus removed cases (n = 19 025) and controls (n = 180 084) that were childless. After removal of childless controls, some cases lacked controls and were thus removed (n = 321).

### Categorization of cases

Cases were classified into five risk categories according to a modification of the National Comprehensive Cancer Network: [17] Low-risk: local clinical stage T1-2, Gleason score ≤6 and serum levels of prostate specific antigen (PSA) <10 ng/mL, intermediate-risk: T1-2, Gleason score 7 and/or PSA 10 to <20 ng/mL, high-risk: T3 and/or Gleason score 8–10 and/or PSA 20 to <50 ng/mL, regionally metastatic disease: T4 and/or N1 and/or PSA 50 to <100 ng/mL in the absence of distant metastases (M0 or Mx) and distant metastases: M1 and/or PSA ≥100 ng/mL.

### Identification of children

We identified singleton and twin births for cases and controls by use of the Multi-Generation Register, a nationwide register created in March 2000 and kept at Statistics Sweden. It includes all subjects born 1932 or later who were alive in 1961, and for each index subject, the parents can be identified [18]. Overall, there is virtually complete parental information on the index subjects, except for those who died before 1991 where the completeness is approximately 50%. Adoptions and other non-biological relationships were not included in the current analysis.

### Swedish twin register

In 2013, PCBaSe 2.0 was linked to the Swedish twin register to obtain zygosity for same-sex twins born to men in the database. The Swedish twin register was established in the 1950^th^: ies and it includes twins born as early as 1886 [19]. Information to the Swedish twin register on twin births is obtained from the National Board of Health and Welfare. For the majority of the same-sex twins, zygosity is determined by questions on physical similarities in childhood, a method that is congruent with DNA based methods in approximately 96% of the cases.

Coverage of twin births and determination of zygosity is generally high although it varies between the years, and for some years in the late 1980-ies the proportion of twin pairs with zygosity determined dropped to 30%.

### Definition of twin fatherhood status

Men in PCBaSe 2.0 were classified as dizygotic twin fathers if they had one or more sets of twins of un-like sex (always dizygotic), or which were dizygotic according to the Swedish twin register. Fathers of same-sex twins with unknown zygosity and those with monozygotic twins according to the Swedish twin register were treated as fathers of singletons in all analysis.

### Fertility treatment

Fertility treatment increase rate of dizygotic twins and this started to affect twinning rates in Sweden in 1990 [20] and we thus choose to only include children born before 1991.

### Assessment of comorbidity

The Patient Register includes all diagnosis from in-patient hospital care in Sweden since 1987 [21], and the register is kept at the National Board of Health and Welfare. Since 1964 the proportion of missing data on main diagnosis has been 1%. We classified cases and controls into four categories of comorbidity by Charlson comorbidity index: 0, 1, 2, 3+ co-morbidities by use of discharge diagnoses in the Patient Register for the ten years preceding the date of diagnosis of prostate cancer [22].

### Assessment of marital status and educational level

The Longitudinal Integration Database for Health Insurance and Labor Market Studies, a nation-wide demographic database kept at Statistics Sweden that includes all subjects above 16 years of age residing in Sweden, was used to obtain information on marital status. Marital status was classified into married (also including registered partnership), divorced, widower, or never married. This database was also used to obtain information on highest attained educational level. Information on marital status and educational level was obtained the year of diagnosis of prostate cancer for the cases and for controls the year of diagnosis of the index case if available. Educational level was available for the years 1990–2009 and if not available for the year of diagnosis, the latest record of educational level was used. We then categorized educational level in four categories; low (nine year grade school), intermediate (high school), high (college), and other.

### Statistical Analysis

Odds ratios (ORs), with 95% confidence intervals (95% CIs), for risk of prostate cancer by dizygotic twin fatherhood status, educational level, marital status and comorbidity were calculated using conditional logistic regression. In stratified analysis, we also evaluated whether the associations were different in the five prostate cancer risk categories. The ORs for risk of prostate cancer by dizygotic twin fatherhood status were not adjusted for educational level, marital status or comorbidity since univariable analysis were non-significant for dizygotic twin fatherhood status, and these factors were evenly distributed between fathers of dizygotic twins and fathers of singletons. All statistical tests were two-sided and the alpha level was 0.05. Calculations were performed with STATA MP/2 version 12.1 (StataCorp LP, College Station, Texas).

### Ethics statement

For PcBaSe, information that data is collected to the NPCR is posted in clinics reporting to the register. Here, the possibility to decline participation is stated, the so-called opt-out principle. The study was approved by the Research Ethical Review Board of Umeå University Hospital.

## Results

There were 1 112 dizygotic twin fathers among 96 301 prostate cancer cases and 4 538 among 378 583 matched controls. There were no substantial differences in educational level, marital status or comorbidity between cases and controls (Table 1), or in men that had fathered dizygotic twins and men that had singletons (Table 2). There was a small difference in the proportion of men diagnosed with prostate cancer as a result of a health examination (i.e. PSA-testing) between men that had fathered dizygotic twins (21%) and men that had fathered singletons (22%), (Table 2) likely due to a calendar effect since fathers of dizygotic twins were diagnosed somewhat more often in the pre-PSA era, Table 3.

**Table 1:** Characteristics of cases and controls in Prostate Cancer database Sweden 2.0.

	Cases (n = 96 301)		Controls (n = 378 583)	
Age	n =	(%)	n =	%
Mean (SD)	71.4	8.9	71.0	9.0
<65	24 681	25.6	92 005	24.3
65 to <75	36 163	37.6	144 055	38.1
≥75	35 457	36.8	142 523	37.6
**Twin fatherhood status**				
Father of digygotic twins	1 112	1.2	4 538	1.2
Father of monozygotic twins	461	0.5	1 717	0.5
Missing zygosity	406	0.4	1 688	0.4
**Marital status** a				
Married	70 812	73.5	270 183	71.4
Divorced	12 355	12.8	54 562	14.4
Widower	11 007	11.4	43 576	11.5
Never married	2 121	2.2	10 262	2.7
Missing data	6	<0.1	0	0
**Educational level** b				
Low	42 328	44.0	173 247	45.8
Intermediate	33 226	34.5	129 733	34.3
High	18 418	19.1	67 175	17.7
Other	2 329	2.4	8 428	2.2
**Comorbidity** c				
0	62 573	65.0	243 148	64.2
1	17 594	18.3	69 575	18.4
2	9 404	9.8	36 963	9.7
3+	6 730	7.0	28 897	7.6

a Marital status determined the same year as diagnosis of prostate cancer in cases (or in the index case of the controls).

b Highest level of education recorded the same year as diagnosis of prostate cancer (or in the index case for the controls) or the latest recorded educational level.

c Classified according to Charlson comorbidity index [22].

**Table 2:** Characteristics of dizygotic twin fathers and fathers of singleton(s) in Prostate Cancer database Sweden 2.0.

	Fathers of dizygotic twins (n = 5 650)		Fathers of singleton(s) (n = 469 234)	
	n =	(%)	n =	(%)
**Age, years**				
Mean (SD)	72.9	(8.7)	71.3	(8.9)
<65	1 045	(18.5)	115 641	(24.6)
65 to <75	2 092	(37.0)	178 126	(38.0)
≥75	2 513	(44.5)	175 467	(37.4)
**Marital status** a				
Married	4 027	(71.3)	336 968	(71.8)
Divorced	750	(13.3)	66 167	(14.1)
Widower	784	(13.3)	53 799	(11.5)
Never married	89	(1.6)	12 294	(2.6)
Missing data	0	0	6	(<0.1)
**Educational level** b				
Low	2 660	(47.1)	212 915	(45.4)
Intermediate	1 856	(32.9)	161 103	(34.3)
High	995	(17.6)	84 598	(18.0)
Other	139	(2.5)	10 618	(2.3)
**Comorbidity** c				
0	3 403	(60.2)	302 318	(64.4)
1	1 128	(20.0)	86 041	(18.3)
2	637	(11.3)	45 730	(9.8)
3+	482	(8.5)	35 145	(7.5)
**Mode of detection** d				
Health examination	229	(20.6)	21 286	(22.4)
Symptoms	523	(47.0)	45 045	(47.3)
Other	44	(4.0)	5 443	(5.7)
Missing data	316	(28.4)	23 415	(24.6)

Fathers of monozygotic twins or twins with unknown zygosity were categorized as fathers of singletons throughout the table.

a Marital status determined the same year as diagnosis of prostate cancer in cases (or in the index case of the controls).

b Highest level of education recorded the same year as diagnosis of prostate cancer (or in the index case for the controls) or the latest recorded educational level.

c Classified according to Charlson comorbidity index [22].

d Information on diagnostic work-up was recorded in NPCR from 2000.

**Table 3:** Demographics and tumor characteristics of prostate cancer cases in Prostate Cancer database Sweden 2.0.

	Fathers of dizygotic twins		Fathers of singleton(s)	
		(n = 1 112)		(n = 95 189)
**Year of diagnosis**				
1991–1995	77	(6.9)	4428	(4.7)
1996–1999	225	(20.2)	17 760	(18.7)
2000–2002	227	(20.4)	18 368	(19.3)
2003–2006	325	(29.2)	31 449	(33.0)
2007–2009	258	(23.2)	23 184	(24.4)
**Risk category** a				
Low-risk	1 212	(21.5)	111 402	(23.7)
Intermediate-risk	1 231	(21.8)	109 896	(23.4)
High-risk	1 528	(27.0)	119 248	(25.4)
Regionally metastatic	525	(9.3)	40 397	(8.6)
Distant metastases	1 008	(17.8)	75 486	(16.1)
Missing data	146	(2.6)	12 805	(2.7)

a Low-risk: T1-2, Gleason score  =  6 and PSA <10 ng/mL. Intermediate-risk: T1-2, Gleason score 7 and/or PSA 10 to <20 ng/mL. High-risk: T3 and/or Gleason score 8–10 and/or PSA 20 to <50 ng/mL. Regionally metastatic disease: T4 and/or N1 and/or PSA 50 to <100 ng/mL in the absence of distant metastases (M0 or Mx). Distant metastases: M1 and/or PSA ≥100 ng/mL.

### Association between dizygotic twin fatherhood status and risk of prostate cancer

**Figure 1:**
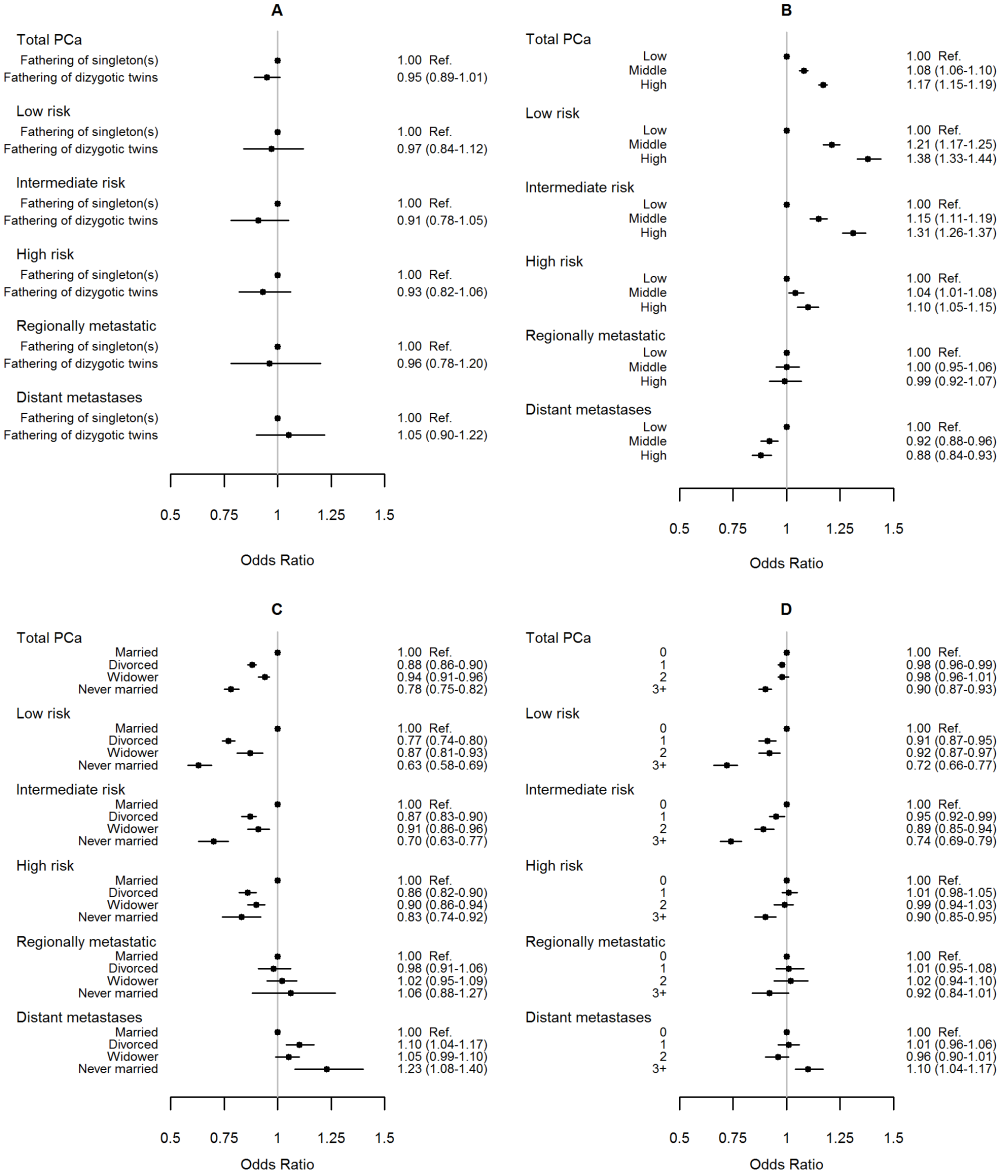
Risk of prostate cancer by A) dizygotic twin fatherhood status, B) educational level, C) marital status, and D) Charlson Comorbidity Index.

In univariable analysis, there was no association between fathering of dizygotic twins and risk of prostate cancer overall compared to fathers of singletons; OR 0.95 (95% CI 0.89-1.01) for total prostate cancer, or to risk of prostate cancer in different risk categories; for low-risk prostate cancer, OR 0.97 (95% CI 0.84–1.12) and metastatic prostate cancer, OR 1.05 (95% CI 0.90–1.22) (Figure 1a).

### Association between marital status, education, comorbidity and risk of prostate cancer

Men with a high educational level had an increased risk of total prostate cancer compared to men with a low educational level, OR 1.17 (95% CI 1.15–1.19) (Figure 1b). Divorced, never married men as well as widowers were at decreased risk of total prostate cancer compared to married men; down to OR 0.78 (95% CI 0.75–0.82) for never married men (Figure 1c). For both marital status and educational level, the associations were stronger for low-risk and intermediate-risk tumors while for metastatic disease, the associations reversed to a decreased risk for men with a high educational level, and to an increased risk for non-married men. Increased comorbidity was associated with a decreased risk of total prostate cancer risk, OR 0.90 (95% CI 0.87–0.93) for CCI3+, compared to CCI 0 (Figure 1d). For metastatic disease, there was an indication of an increase in risk, OR 1.10 (95% CI 1.04–1.17) for CCI 3+.

## Discussion

In this nationwide, population-based case-control study male fertility measured as fathering of dizygotic twins was not associated with increased risk of prostate cancer. The associations between educational level, marital status and comorbidity and risk of prostate cancer are likely reflections of differences in uptake of PSA testing.

### Strengths and limitations

Our study was large, population-based and included detailed information on tumor characteristics for cases, zygosity for same-sex twins fathered by men in the database, as well as socioeconomic factors and comorbidity for cases and controls. By linkage to the Swedish twin register, we obtained zygosity for almost 80% of the twins. However, due to missing data on zygosity for some twins in the Swedish twin register there was an underestimation of dizygotic twins in our dataset (some same sex twins remained un-classified and were treated as singletons in all analysis). The proportion of un-classified same-sex twins was similar between cases and controls (20.5% vs 21.3%), making the misclassification non-differential, and it should thus have decreased the precision in our analysis, but should not have affected our risk estimates. Furthermore, we do not know at what level of impaired male fertility the likelihood of dizygotic twinning is reduced and we are unaware of any data of the possible correlation between dizygotic twinning and male androgen levels.

Rate of dizygotic twinning is influenced by heredity, parity, fertility, and age of the mother, while monozygotic twinning occurs randomly. The ratio of dizygotic twinning has been used to estimate trends in fertility in populations [9], [10]. Also, couples with a longer time to pregnancy are also less likely to have twins with a stronger effect for dizygotic twins [23], [24]. With regard to male fertility; in a study on semen parameters in 37 fathers of dizygotic and 15 fathers of monozygotic twins and 349 fathers of singletons as controls, men with twins had better sperm quality then fathers of singletons [25]. Also, in a study on testicular cancer, the ratio of dizygotic twinning was clearly reduced in subfertile men that later developed testicular cancer [11]. These findings give support that this study design is efficient for studying the relationship between fertility and risk of cancers that are associated with fertility.

Previous studies on the association between fertility and prostate cancer have compared risk of prostate cancer in fathers with that of childless men, under the assumption that fathers on average have higher levels of androgens than the subgroup of childless men that are sub/infertile [5]. In some small studies on fatherhood status, the association has been inconclusive [26]–[30] but in three large register-based studies, fathers have had an increased risk of prostate cancer [1]–[3]. However, in all these studies confounding may be present since fathering of children is affected by other factors except male fertility, such as fertility of the partner, presence of female partner and wish to have children [31].

Two studies have examined the risk of prostate cancer in infertile men; one Swedish study reported that infertile men had half the risk of prostate cancer compared to fertile men [32] while a US study showed that infertile men had a two-fold increase in risk of high-grade cancer (Gleason score 8–10) [33]. However, both these studies were limited in size.

Dizygotic twinning is increased after use of fertility treatment such as in vitro fertilization and if many of the twins in our study were a result of fertility treatment then dizygotic twinning would be a marker of *decreased* instead of *increased* fertility, as in our hypothesis. In vitro fertilization was introduced 1981 in Sweden but twinning rates did not increase markedly until 1990 [20], and in order to avoid this mis-classification we removed children born 1991 or later from all analysis.

## Conclusions

Our results do not support an association between male fertility and prostate cancer risk.
